# The Responsiveness of Bee Venom Phospholipase A2 on Regulatory T Cells Correlates with the CD11c^+^CD206^+^Population in Human Peripheral Blood Mononuclear Cells

**DOI:** 10.3390/toxins13100717

**Published:** 2021-10-10

**Authors:** Heejin Jo, Hyunjung Baek, Seon-Young Park, Bonhyuk Goo, Woo-Sang Jung, Hyunsu Bae, Sang-Soo Nam

**Affiliations:** 1 Chaum Life Center, Department of Korean Medicine, CHA University School of Medicine, Seoul 06062, Korea; kmdrjin@gmail.com; 2 Department of Physiology, College of Korean Medicine, Kyung Hee University, Seoul 02453, Korea; bguswjd@naver.com (H.B.); psys12@naver.com (S.-Y.P.); 3 Department of Acupuncture & Moxibustion, Kyung Hee University Hospital at Gangdong, Seoul 05278, Korea; goobh99@naver.com; 4 Department of Cardiology and Neurology, College of Korean Medicine, Kyung Hee University, Seoul 02447, Korea; wsjung@khu.ac.kr

**Keywords:** regulatory T cell (Treg), bee venom phospholipase A2 (bvPLA2), human peripheral blood mononuclear cell (hPBMC), CD206, mannose receptor

## Abstract

Bee venom phospholipase A2 (bvPLA2) has been reported to have therapeutic effects such as neuroprotection, anti-inflammation, anti-nociception, anti-cancer properties, caused by increasing regulatory T cells (Tregs). The mechanism of Tregs modulation by bvPLA2 has been demonstrated by binding with the mannose receptor, CD206 in experimental models of several diseases. However, it remains unknown whether this mechanism can also be applied in human blood. In this study, we collected peripheral blood samples from healthy donors and analyzed the percentages of monocyte-derived dendritic cells with CD206 (CD206^+^ DCs) before expansion, the proportion of Tregs, and the subpopulations after expansion treated with bvPLA2 or PBS using flow cytometry and the correlations among them. The percentage of Tregs tended to be higher in the bvPLA2 group than in the control group. There were significant positive correlations between the CD206 population in hPBMC and the proportions of Tregs treated with bvPLA2, especially in the Treg fold change comparing the increase ratio of Tregs in bvPLA2 and in PBS. These findings indicate that bvPLA2 increased the proportion of Tregs in healthy human peripheral blood and the number of CD206^+^ DCs could be a predictor of the bvPLA2 response of different individuals.

## 1. Introduction

Bee venom has been used to treat severe pain and inflammatory diseases such as rheumatoid arthritis (RA) in Korean medicine [1,2]. The range of applications has increased, with recent studies reporting its therapeutic effects in amyotrophic lateral sclerosis (ALS) [3,4,5], Parkinson’s disease (PD) [6], Alzheimer’s disease (AD) [7], multiple sclerosis (MS) [8], cancer [9], and atherosclerosis [10]. The mechanism of those therapeutic effects has been investigated with bee venom phospholipase A2 (bvPLA2), one of the major components of the venom, making up about 10 to 12% of bee venom and the second largest proportion after mellitin [11]. BvPLA2 has been reported to have an anti-inflammatory effect on acetaminophen-induced acute liver injury [12], cisplatin-induced acute kidney injury [13], ovalbumin-induced allergic airway inflammation [14,15], radiation-induced acute lung inflammation [16], house dust mites-induced atopic dermatitis [17,18], anti-nociceptive effect on oxaliplatin-induced neuropathic pain in animal models [19], neuroprotective effect on PD [20,21], AD [22,23], anti-cancer effects [24,25], and anti-viral effects [26].

These effects are explained primarily by the relationships between bvPLA2, CD206, and regulatory T cells (Tregs). BvPLA2 is specifically recognized by CD206. This is because of the structural complexity of bvPLA2, which is a glycoprotein consisting of oligosaccharide structures containing mannose residues [27,28], unlike the others such as the snake and pancreatic PLA2, which consist of simple proteins [29,30]. Treatment with bvPLA2 has been shown to activate and increase the number of CD4^+^CD25^+^Foxp3^+^ Tregs in pathological animal models. Tregs, a subset of CD4^+^ T cells with immunosuppressive effects, are important in the regulation of peripheral CD4^+^ T cells in regional inflammatory injury, and in maintaining physiological immune homeostasis by managing many aspects of the immune reaction [31]. They suppress a wide range of immune cells, including CD4^+^ T cells, CD8^+^ T cells, B cells, macrophages, mast cells, natural killer (NK) cells, and dendritic cells (DCs) [32,33]. In the absence of Tregs, bvPLA2 does not have the aforementioned therapeutic effects [12,14,16,17,19,22]. Tregs do not exert a suppressive role in a CD206-deficient model, in spite of bvPLA2 stimulation [13,15,18,20,21,23]. This study is the first attempt to examine that the previously reported relationship among bvPLA2, CD206, and Tregs would be also applied in healthy human peripheral blood.

In peripheral blood, the proliferation of Tregs under inflammation caused by a specific stimulus is induced by dendritic cells (DCs) which present antigens to CD4^+^ T cells. The cell surface receptors through which DCs detect the antigen, bvPLA2, are thought to be CD206 [34,35]. However, to our knowledge, it has not been investigated whether the amount of DCs in healthy human peripheral blood would influence on the degree of Tregs expansion by bvPLA2. Tregs are subdivided functionally and phenotypically and the frequency of those subsets are different from ages, pathological conditions [36]. To our knowledge, it has not been studied how Treg subsets expansion would be affected by bvPLA2.

In this study, we hypothesized that bvPLA2 also induces Treg activation and accretion in healthy human peripheral blood that the amount of monocyte-derived CD206-positive DCs in the peripheral blood influences the extent of Treg reaction to bvPLA2. We also investigated what changes happen in Tregs differentiation. The results would support that bvPLA2 has immunomodulatory effects and suggest the possibility of utilizing CD206 in human peripheral blood to predict Tregs response to bvPLA2, an important component of bee venom acupuncture.

## 2. Results

### 2.1. Effect of bvPLA2 Treatment on the Treg Expansion of hPBMCs

**Figure 1:**
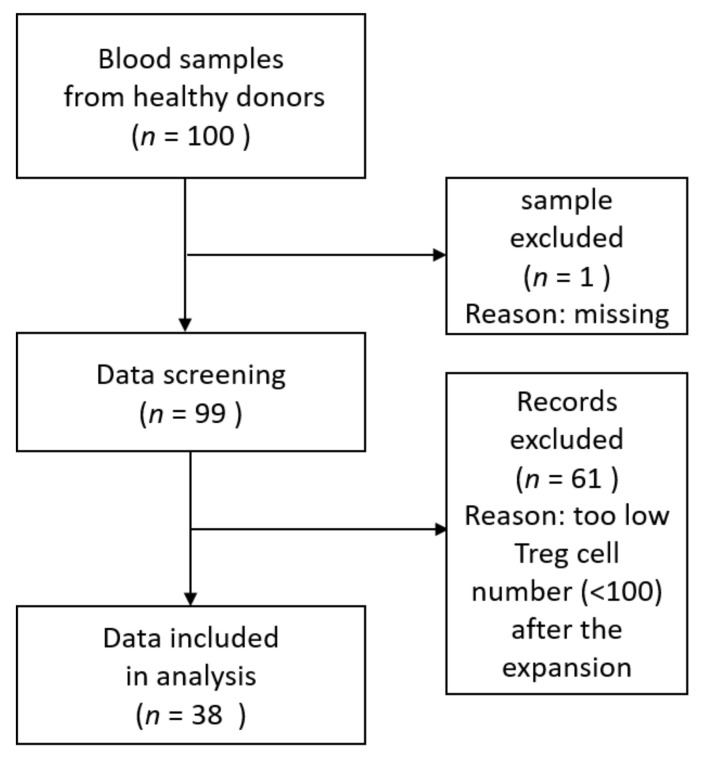
Data selection process.

To confirm whether bvPLA2 influences Tregs in hPBMCs or not, the percentages of CD4^+^CD25^+^CD127^low^Foxp3^+^ Tregs (Tregs) after expansion treated with bvPLA2 or PBS as control were analyzed and compared. Among the 100 blood samples (72 women and 28 men, mean age 34.97 ± 12.78), one sample was missed during the screening process. Sixty-one records were excluded because of low Treg cell numbers, defined as less than 100 after Treg expansion in the PBS-treated control group. Only thirty-eight samples from the healthy donors (30 women and 8 men) were included in the data analysis (Figure 1). The average age of the donors included was 34.71 ± 1.95 and the age range was from 20 to 59.

**Figure 2:**
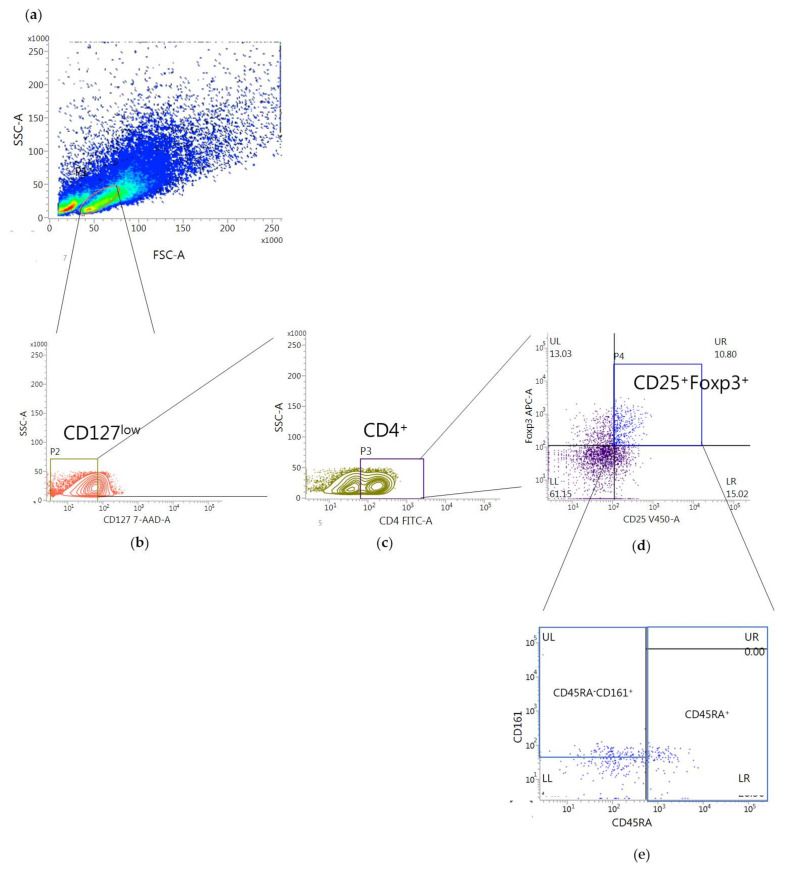
CD4^+^CD25^+^CD127^low^Foxp3^+^ Tregs and its subsets in hPBMCs were analyzed by flow cytometry after the Treg expansion. (**a**) The lymphocytes in hPBMCs after Treg expansion treated with bvPLA2 or PBS were gated for Tregs (**b**,**c**). (**d**) The percentages of CD25^+^Foxp3^+^ cells in the CD4+CD127^low^ fraction were calculated. (**e**) Tregs were gated for analysis of Treg subsets; CD4^+^CD25^+^CD127^low^Foxp3^+^CD45RA^+^ naïve Tregs (n-Tregs) and CD4^+^CD25^+^CD127^low^Foxp3^+^CD45RA^−^CD161^+^ inflammatory Tregs (i-Tregs). The percentages of CD45RA^+^ or CD45RA^−^CD161^+^ populations in the CD4^+^CD25^+^CD127^low^Foxp3^+^ Tregs fraction were calculated.

**Figure 3:**
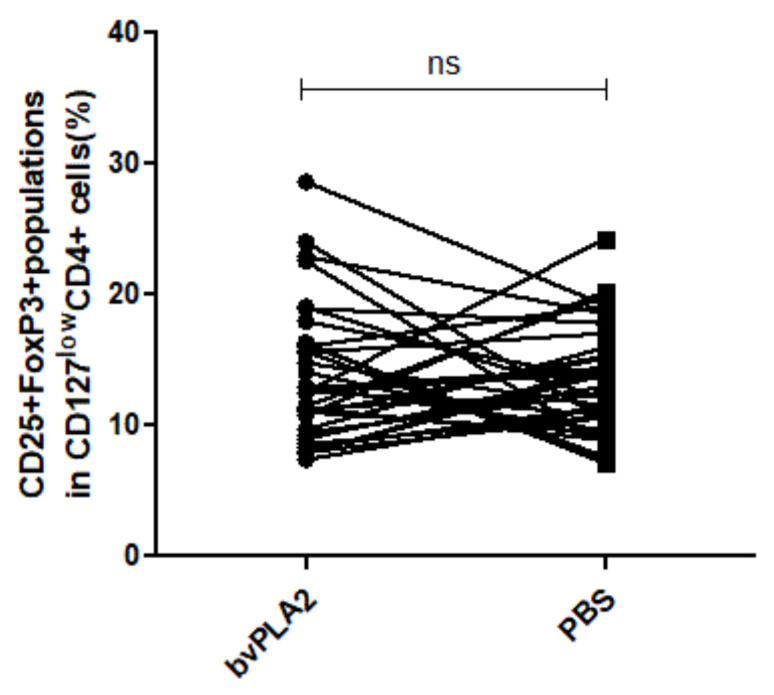
The percentages of total expanded Tregs were compared between the bvPLA2 and the PBS groups. When hPBMCs were treated with bvPLA2, the percentage of Tregs after the expansion was higher than that in the PBS control group, without significant difference (mean difference = 0.3779, *p* = 0.7138), ns: not significant.

Figure 2 demonstrates the gating strategy to identify the frequency of Tregs. The percentage of Tregs in human PBMCs treated with bvPLA2 was higher than that in control cells treated with PBS although the difference was not significant (mean difference = 0.3779, *p* = 0.7138) (Figure 3).

### 2.2. Effect of bvPLA2 Treatment on the Treg Subsets after Expansion in hPBMCs

**Figure 4:**
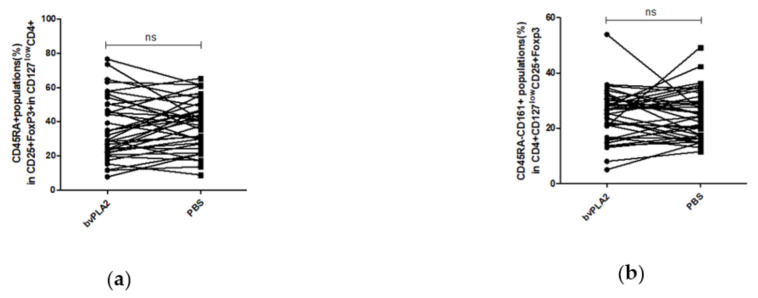
The proportions of Tregs subsets were compared between the bvPLA2 group and the PBS control group. (**a**) The percentage of n-Tregs was higher in the bvPLA2 group than in the control (mean of differences = −1.834, *p* = 0.3934). (**b**) The percentage of i-Tregs was also higher, or nearly similar, in the bvPLA2 group than in the control group (mean of differences = 0.1418, *p* = 0.9238), ns: not significant.

To investigate the effect of bvPLA2 on the increased Treg in hPBMCs, the percentages of CD45RA^+^ population and CD45RA^−^CD161^+^ population in the CD4^+^CD25^+^CD127^low^Foxp3^+^ Tregs were further analyzed (Figure 2e) as Treg sub-populations, naïve Tregs, and proinflammatory Tregs respectively. Although there were no significant differences, the percentage of the CD4^+^CD25^+^CD127^low^Foxp3^+^CD45RA^+^ naïve Treg cells (n-Tregs) was higher in the bvPLA2 group than in the control (mean of differences = −1.8340, *p* = 0.3934) (Figure 4a) and the percentage of the CD4^+^CD25^+^CD127^low^Foxp3^+^CD45RA^−^CD161^+^ inflammatory Tregs (i-Tregs) in the bvPLA2 group was similar with that in the control group (mean of differences = −0.1418, *p* = 0.9238) (Figure 4b).

### 2.3. Correlations between Tregs after Treated with bvPLA2 or PBS and CD11c^+^CD206^+^ DCs in hPBMCs

**Figure 5:**
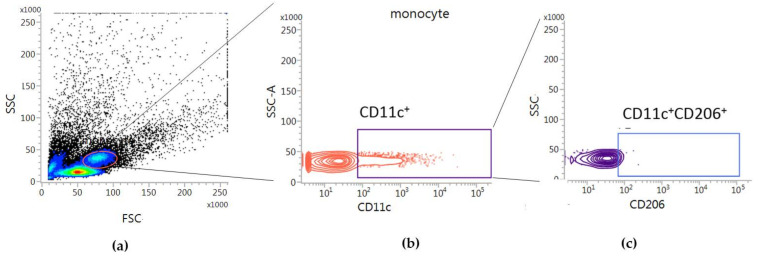
CD206^+^ DCs population in hPBMCs were analyzed by flow cytometry. The monocytes in human peripheral blood (**a**) were gated for CD11c^+^ DCs (**b**). (**c**) The percentages of CD206^+^ population in the CD11c^+^ DCs fraction were calculated.

Figure 5 illustrates the gating strategy to identify the dendritic cells which contains CD206 in hPBMCs. The ratio between the percentage of Tregs in the bvPLA2 and that in the control group was defined as Treg fold change.

**Figure 6:**
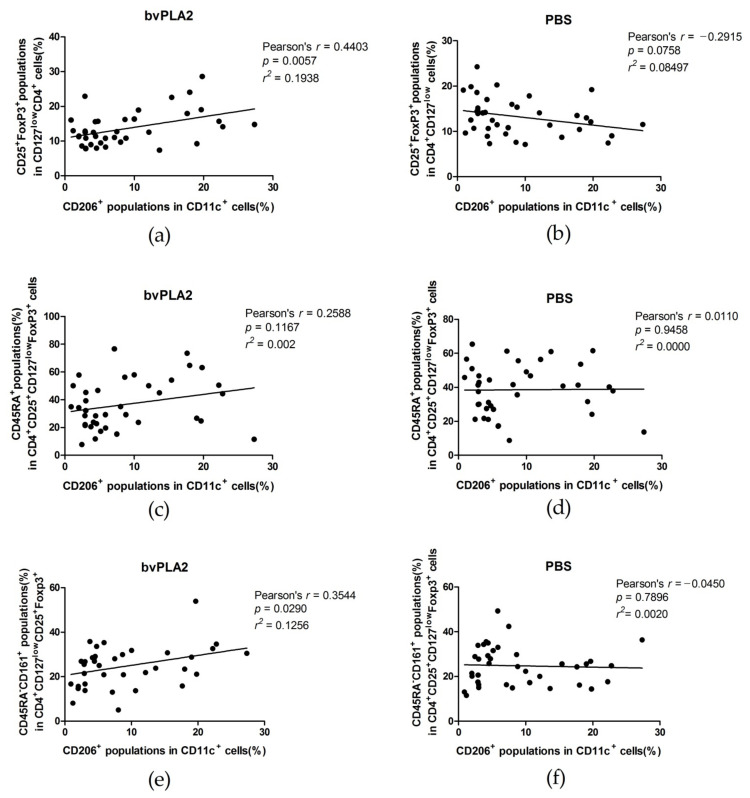
Correlation and linear regression analysis were conducted between the frequency of CD206^+^ DCs in hPBMCs and the frequency of Tregs and the subsets after treatment with bvPLA2 and PBS. (**a**) The relationship between the CD206^+^ populations in hPBMC before expansion and the percentage of Tregs after expansion treated with bvPLA2 was analyzed. There was a significant low positive correlation (Pearson’s *r* = 0.4403, *p* = 0.0057). (**b**) There was a negligible negative correlation after treatment with PBS as the control, without significance (*p* = 0.0758). (**c**) The correlations between the CD206^+^ population in hPBMC before expansion and the percentage of n-Tregs after expansion were found to be negligible, without significance in both the bvPLA2 (Pearson’s *r* = 0.2588, *p* = 0.1167) (**d**) and PBS groups (Pearson’s *r* = 0.011, *p* = 0.9458). (**e**) There was a significant low positive correlation between the percentage of i-Tregs in bvPLA2 and the CD206^+^ DCs in hPBMCs (Pearson’s *r* = 0.3544, *p* = 0.0295). (**f**) The correlation between the percentage of i-Tregs in the PBS group and the CD206^+^ population in hPBMCs was negligible (Pearson’s *r* = −0.045, *p* = 0.7896).

**Figure 7:**
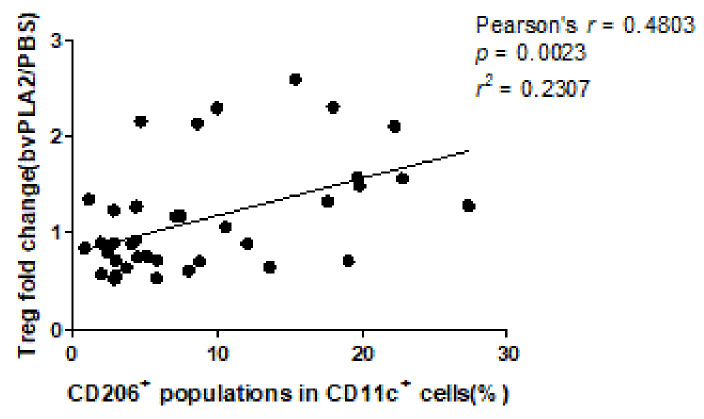
Correlation and linear regression analysis were conducted between CD206^+^ DCs in hPBMCs and Treg fold change. Treg fold change had a significant low positive correlation with the CD206^+^ population in hPBMCs (Pearson’s *r* = 0.4803, *p* = 0.0023).

We analyzed the correlations of the percentage of CD11c^+^CD206^+^ DCs in PBMCs with the percentages of Treg cells and its subsets, n-Tregs and i-Tregs, in the bvPLA2 and the PBS control group. The correlation of CD206^+^ DCs with Treg fold change was also calculated. We expected the results would show whether CD206^+^ DCs in PBMCs could be a factor related to the responsiveness of bvPLA2 on Tregs in human peripheral blood. There were significant positive correlations in the cases of the percentage of Tregs in bvPLA2 (Pearson’s *r* = 0.4403, *p* = 0.0057), the percentage of i-Tregs in bvPLA2 (Pearson’s *r* = 0.3544, *p* = 0.0290) (Figure 6), and Treg fold change (Pearson’s *r* = 0.4803, *p* = 0.0023) (Figure 7).

## 3. Discussion

Appropriate doses of bvPLA2 do not induce toxicity [37], but can produce therapeutic effects via increasing Tregs in experimental models [12,13,15,16,17,19,20,22,23,24,38]. However, bvPLA2 can exert this role affecting Tregs to suppress or regulate the immune response only in the presence of CD206 [13,15,18,20,21,23] as it is recognized by this mannose receptor. In clinical practice, it has been observed that the same intensity of treatment can produce different effects depending upon an individual’s characteristics, and the study on these characteristics and their correlation with the effects, or predictors of effectiveness, is needed in order to implement personalized medicine. We confirmed that the immunomodulatory effect of bvPLA2, a major component of bee venom, would also act on the healthy human peripheral blood. Although it is not significant, bvPLA2 increased the proportion of Tregs, n-Tregs, and i-Tregs, in hPBMCs from healthy donors compared with treatment with PBS. Moreover, the data showed that Treg fold change to compare the difference of Treg expansion in hPBMC between bvPLA2 and PBS had a positive correlation with the CD206^+^ DC population in peripheral blood. This suggests that the population of the CD206^+^ DC would be a predictor for the degree of immune response to bvPLA2 in human peripheral blood.

In this study, CD4^+^CD25^+^CD127^low^Foxp3^+^ cells were used to detect functionally active Tregs [39]. Consequently, the sample number included in the analysis was smaller than expected; less than half of the healthy donors enrolled. This could be caused by the marker we used for identifying Tregs, which is the most specific one. CD4^+^CD25^+^ Tregs strongly suppressed the immune response [40]. Foxp3, a transcription factor, is critical in the regulation of Treg function and development and identification of Foxp3, an intracellular molecule, requires cell fixation and permeabilization [41,42,43]. Lack of CD127, the α chain of the IL-7 receptor, is used in combination with CD25 to identify human Tregs from conventional CD4^+^ T cells [44].

It is controversial whether the frequency of Tregs reflects the severity of diseases such as RA [45,46]. Sometimes, increased Tregs at the site of inflammation of a pathological model or a patient did not function properly [45]. Tregs are believed to increase at an early stage of disease, to inhibit disease progression, and to decrease at an irreversible severe stage, producing less inflammation [47,48,49]. Increasing the number of peripheral Tregs was one of the key factors in the mechanism of and clinical response to new therapeutic material candidates modulating the immune system. Previous reports have indicated that peripheral-blood-derived Tregs from healthy donors were present at a higher frequency than those in a pathological model or patients [45,46]. The condition of the study subjects could therefore affect the degree of change induced by bvPLA2, resulting in findings with no statistical significance.

Treg subpopulations were further analyzed. It was observed that, although the mean differences were not significant, n-Tregs were increased by bvPLA2 compared to PBS, and i-Tregs were increased to a lesser extent than n-Tregs. It is possible that Tregs i-n hPBMCs could be developed and differentiated better by bvPLA2, preparing n-Tregs for an immediate reaction or differentiation with any antigens or cytokines, and i-Tregs for immediate cytokine secretion when needed.

CD206 is known to recognize bvPLA2 by binding with it [30]. CD206 can be found on both macrophages and DCs, but only CD206 on DCs can activate the immune suppressive program of bvPLA2 [15]. Under specific conditions, DCs are derived from monocytes and play a role in presenting antigens to other cells [50]. BvPLA2 binds to CD206 on DCs, resulting in the release of prostaglandin E2, and increasing the number of Tregs [51]. In previous studies, the immune modulation effect of bvPLA2 on Tregs has been reported to be mediated by mannose receptors [13,15,18]. We expected that the extent of Treg increase caused by bvPLA2 would be dependent on the CD206 positive DC population in human peripheral blood. The results showed a low positive correlation that the percentages of Tregs and n-Tregs and i-Treg subsets were significantly increased in the bvPLA2 group and not in the control group, corresponding with the CD206^+^ DC population. Treg fold change—the ratio between the bvPLA2 group and the PBS group—confirmed a quantitative difference in the expression of Tregs, with the highest correlation being with the proportion of CD206^+^ DCs. It is possible that more Tregs would be induced under the same amount of bvPLA2 stimulation if more CD206 existed. These results could suggest that CD206 could be a marker to predict individual susceptibility to bvPLA2 treatment.

## 4. Conclusions

This observation confirms that the regulatory effect of bvPLA2 on Tregs is also applied to hPBMC and suggests that the proportion of CD206^+^ DC in human peripheral blood could be a marker to predict the susceptibility of individuals to the application of bvPLA2, one of the major bioactive components of bee venom. Future research should be conducted in larger samples and focus on how to apply it for practical use.

## 5. Materials and Methods

### 5.1. Isolation of Human Peripheral Blood Mononuclear Cells (hPBMCs)

Peripheral blood (16 mL) was obtained from 100 healthy volunteers (72 women and 28 men, mean age 34.97 ± 12.78 range from 20 to 67) enrolled from May 2018 to August 2018, who had not experienced any inflammatory disease within the past month. All donors provided the informed consent to participate in this study. The study protocol was approved by the Ethics Committee of Kyung Hee University Korean Medicine Hospital (KOMCIRB-170818-BR-033).

Each 8 mL of peripheral blood was collected in a BD Vacutainer CPT tube (BD Bioscience, San Jose, CA, USA) and mixed. After centrifugation for 20 min at 2000 rpm, the peripheral blood monocyte layer weas moved into a new tube and washed with sterile phosphate-buffered saline (PBS). Cells were resuspended in ex vivo media (Lonza, Walkersville, MD, USA) containing 10% human serum (Sigma, ST. Louis, MO, USA) and 1% penicillin and streptomycin (Gibco, Gaithersburg, MD, USA). After adding 10% DMSO (Sigma-Aldrich, St. Louis, MO, USA), the cells were frozen and preserved.

Before being treated, the cells were thawed quickly in a water bath at 37 °C and washed with sterile PBS. Cells were resuspended in the growth media and used for further experiments.

### 5.2. Regulatory T Cell Expansion

After counting the cells, we diluted the samples to a concentration of 1 × 106 cells/mL for expansion of human regulatory T cells. One hundred microliters (1 × 105 cells) of hPBMC and 20 uL of CD3/CD28 MACSiBead were transferred to each well of a U-bottom 96-well plate, to which were added 100 uL of the medium and 500 U of rhIL-2 (Peprotech, Rocky Hill, NJ, USA, approved on 10 November 2017).

Each sample in the intervention group was treated with 0.4 ug/mL of bee venom PLA2 (bvPLA2, Sigma-Aldrich, St. Louis, MO, USA) and the control group received the same amount of PBS. Samples were incubated for 72 h at 37 °C under 5% CO_2_.

### 5.3. Flow Cytometric Analysis

PBMCs were transferred to a new tube containing 100 ul of BD FACS stain buffer (BD Bioscience, San Jose, CA, USA) and stained with a combination of antibodies: BV510-CD11c, PE-CD206 for analysis of monocytes or FITC-CD4, APC-Cy7-CD3 for analysis of lymphocytes. After resuspension and incubation at 4 °C for 30 min, samples were washed with the stain buffer.

To measure changes in the number of regulatory T cells, the following surface antibodies were used: FITC-CD4, BV421-CD25, PE-CD161, PE-Cy7-CD45RA, BB700-CD127. After staining with the surface antibodies, samples were washed with stain buffer. Foxp3/Transcription factor fixation/permeabilization concentrate and diluent (eBioscience, San Diego, CA, USA) were mixed in a 1:3 ratio and added to each samples. Samples were incubated for 30 min at 4 °C, and the same amount of permeabilization buffer (1×, eBioscience, San Diego, CA, USA) was added and centrifuged for five minutes at 400 *g*. The supernatant was removed and the diluent of the AF647-Foxp3 antibody was added. Samples were incubated at 4 °C for 30 min and washed with permeabilization buffer.

All FACS data were acquired using a FACSLyric™ (BD Biosciences, San Jose, CA, USA) and analyzed using FACSuite software (BD Biosciences, San Jose, CA, USA). Measurements of the population of CD11c^+^CD206^+^ DC cells were obtained by using a gating strategy on peripheral blood samples stained with anti-CD11c and anti-CD206 to detect the specific surface markers of monocytes (Figure 5). Peripheral blood mononuclear cells (PBMCs) treated with the Treg expansion kit were gated for CD127^low^ and CD4^+^ T cells, and the percentage of CD4^+^CD25^+^CD127^low^Foxp3^+^ was identified in the Tregs (Figure 2). Naïve Tregs were identified as CD45RA^+^ in CD4^+^CD25^+^CD127^low^Foxp3^+^ Tregs, and inflammatory Tregs were identified as CD45RA^−^CD161^+^ in CD4^+^CD25^+^CD127^low^Foxp3^+^ Tregs (Figure 2e).

### 5.4. Statistical Analysis

All data are presented as mean ± S.E.M. A paired t-test was performed to compare the differentiation of Treg cells in the bvPLA2 group with that in the control group. Correlation analyses and linear regression analyses were performed using Pearson correlation coefficients [52]. Statistical analysis was performed using GraphPad Prism version 5.0 (GraphPad software Inc., San Diego, CA, USA). Results were considered to be statistically significant when *p* values were less than 0.05.

## Data Availability

Data is available upon reasonable request.
